# Sociotechnical influences on the adoption and use of AI-enabled clinical decision support systems in ophthalmology: a theory-based interview study

**DOI:** 10.1186/s12913-025-13620-w

**Published:** 2025-10-22

**Authors:** Insa Schaffernak, Julia Cecil, Anne-Kathrin Kleine, Eva Lermer

**Affiliations:** 1 Department of Business Psychology, Technical University of Applied Sciences Augsburg, An der Hochschule 1, 86161 Augsburg, Germany; 2 Center for Leadership and People Management, LMU Munich, Geschwister-Scholl-Platz 1, 80539 Munich, Germany

**Keywords:** AI-CDSS, Ophthalmology, Qualitative content analysis, Interviews, NASSS, AI chasm, AI adoption, Abandonment, Sociotechnical framework

## Abstract

**Background:**

Artificial intelligence (AI) has the potential to ease the increasing workload in ophthalmology by supporting ophthalmologists’ clinical decision-making. However, despite regulatory approvals, the adoption and use of AI-enabled clinical decision support systems (AI-CDSS) in ophthalmology remains limited. Critical obstacles that innovative healthcare technologies such as AI-CDSS face on their path to widespread clinical use are nonadoption and abandonment by their intended users, which prevent broader dissemination and real clinical benefit. This study explores how to overcome nonadoption and prevent abandonment of ophthalmic AI-CDSS by identifying ophthalmology professionals’ requirements for adoption and continued use of such tools in clinical practice.

**Methods:**

We conducted semi-structured interviews with 22 ophthalmology professionals from Germany, Austria, and Switzerland, representing a range of professional roles, clinical settings, and extent of AI-CDSS experience. To explore sociotechnical factors shaping ophthalmology professionals’ adoption decisions and their ability to derive added value from ophthalmic AI-CDSS, we conducted a qualitative content analysis combining deductive and inductive coding. The Nonadoption, Abandonment, Scale-Up, Spread, and Sustainability (NASSS) framework deductively guided the development of higher-level code categories, representing seven sociotechnical domains relevant for the implementation of healthcare technologies. These were further refined into subcategories through inductive coding of the interview material.

**Results:**

Most participants expressed general openness to ophthalmic AI-CDSS. However, actual adoption decisions and the ability to derive clinical value from these tools were shaped not just by individual attitudes but also by a range of other sociotechnical influences. Specifically, we inductively identified 29 code categories, representing sociotechnical influences and requirements from all seven NASSS domains, including technological, user, organizational, and societal aspects. Our findings also suggest that while many sociotechnical influences and challenges are shared between AI-based and traditional healthcare technologies, a key distinction of (ophthalmic) AI-CDSS lies in the users’ psychological appraisal of such tools.

**Conclusions:**

Our findings highlight the complex and context-specific nature of integrating AI-CDSS into ophthalmic practice. This study also informs AI and healthcare researchers on the applicability of the NASSS framework for studying AI implementation and provides actionable insights for AI developers and implementers aiming to address user needs more effectively.

**Supplementary Information:**

The online version contains supplementary material available at 10.1186/s12913-025-13620-w.

## Background

Ophthalmology—the medical specialty dedicated to eye care—is under growing strain due to the increasing prevalence of eye diseases and demographic changes [1, 2]. An aging population and the rising prevalence of diabetes contribute to a higher incidence of age- and diabetes-related eye diseases, which require frequent monitoring and treatment appointments. At the same time, many ophthalmologists in developed countries are expected to retire in the near future, outpacing the number of new specialists entering the field [3]. For example, by 2035, the ophthalmic workforce in the United States is expected to fall short by 30%—a deficit that is projected to be more severe than in many other medical specialties [4]. The growing disparity between the demand for eye care and the available resources threatens the timely detection and treatment of eye diseases, many of which cause severe vision impairments if not addressed promptly [2, 5]. Consequently, efficient clinical decision-making becomes more and more important to manage ophthalmology professionals’ expanding workload and to ensure adequate eye care for all patients.

The use of artificial intelligence (AI) in ophthalmology may alleviate this burden by supporting and streamlining clinical decision-making. In this field, medical imaging techniques, such as optical coherence tomography (OCT) and fundus photography, are central for diagnostic and treatment decisions for common eye diseases such as age-related macular degeneration (AMD), diabetic retinopathy (DR), and glaucoma [6, 7]. AI’s ability to analyze and classify such images positions it as a promising tool to support ophthalmologists’ decision-making in various image-based tasks such as screening, diagnosis, prognosis, and treatment planning [2, 6]. Ophthalmic AI-enabled clinical decision support systems (AI-CDSS) are designed to provide ophthalmologists with additional insights into retinal images, with the goal to enhance the accuracy and timeliness of disease detection and clinical decisions. These systems process retinal features beyond human perception, enabling the detection of subtle pathologies and monitoring of their progression over time [8]. Some ophthalmic AI-CDSS also offer automated diagnoses, disease grading, or treatment recommendations [2, 8, 9]. Comparative studies indicate that many AI models perform at or above expert ophthalmologist levels in diagnosing eye diseases [2].

Despite such promising applications, however, the clinical adoption and use of ophthalmic AI-CDSS remain limited [7, 10]. Several AI-based devices for ophthalmic applications have been approved by the United States Food and Drug Administration (e.g., IDx-DR, AEYE-DS, EyeArt), or received Conformité Européene marking in the European Economic Area (e.g., RetinAI, RetInSight, Altris AI) [11]. Moreover, an exponentially growing volume of research on the development and evaluation of AI models for ophthalmology suggests that more applications and approvals will follow^1^. So far, however, this progress has not translated into widespread clinical use, illustrating the so-called *AI chasm* [7, 10]. The AI chasm in healthcare refers to the gap between the development and clinical implementation of AI applications, where promising tools fail to achieve widespread use and meaningful clinical benefit [12].

In order for ophthalmic AI-CDSS to provide real-life clinical benefit, we need to understand how they can be successfully translated into ophthalmic practice. To help bridge the AI chasm in ophthalmology, this interview study examined which factors shape ophthalmology professionals’ adoption and use of AI-CDSS, providing insights into user needs, implementation challenges, and strategies for achieving added value in clinical practice.

### Nonadoption and abandonment as obstacles to widespread use

The Nonadoption, Abandonment, Scale-up, Spread, Sustainability (NASSS) framework [13] identified *nonadoption* and *abandonment* as key obstacles new healthcare technologies face after their development and regulatory approval. Nonadoption, meaning clinicians’ decision not to start using a new technology, prevents the necessary first step towards use in clinical practice. Yet, even when initially adopted, many technologies are later abandoned if perceived as offering only limited added value in clinical practice [13–15]. Nonadoption and abandonment by individual users or healthcare institutions therefore keep the innovation from becoming widely used and providing meaningful impact at scale.

Clinicians’ individual attitudes, experiences, and behaviors substantially influence whether a new technology is embraced and retained in clinical practice [13, 14], making them central actors in overcoming the AI chasm. Depending on the dynamics of the healthcare institution and their position within it, clinicians may influence institutional decision-making by advocating for or against the introduction of AI-CDSS, or directly act as gatekeepers themselves, who decide whether to start or stop using AI-CDSS in their daily routine [16].

The phenomenon that newly developed healthcare innovations are not—or only after a significant delay—transferred into routine practice on a large scale is not unique to AI-based technologies [17, 18]. However, unique characteristics of AI, such as the opacity of machine learning algorithms, have prompted calls for research that specifically investigates how AI applications can be effectively embedded into clinical practice [19–21].

### The role of sociotechnical factors for AI-CDSS adoption and use

Implementation research highlights that adoption and use of healthcare innovations are not solely determined by their technical performance but also shaped by a range of social, organizational, and contextual factors [17], which together are termed sociotechnical factors. Sociotechnical frameworks provide a valuable lens for examining how ophthalmic AI-CDSS can be successfully translated from development into clinical practice, as they acknowledge factors at different systemic levels such as the micro level (e.g., clinicians’ attitudes), meso level (e.g., healthcare institutions’ infrastructure), and macro level (e.g., policies).

A sociotechnical perspective is useful for understanding adoption decisions, where professionals evaluate whether AI-CDSS fit into their clinical practice. A few, primarily survey-based studies have explored ophthalmology professionals’ perceived benefits and risks regarding ophthalmic AI. These studies found that while clinicians recognize its potential to improve efficiency and patient care, they are also concerned about reliability, trustworthiness, commercialization, potential disruptions to workflows, patient relationships, and professional identity [22–27]. Clinicians’ perceived benefits and risks are important considerations, but they represent only one part of a larger, dynamic decision-making process. As a recent cross-disciplinary review showed, AI adoption in healthcare is influenced by a complex set of interacting sociotechnial factors, including budget constraints, user attitudes, and adoption readiness of healthcare institutions [28].

Beyond adoption, sociotechnical factors also determine whether newly implemented AI-CDSS deliver meaningful value in clinical practice [13, 29]. Sociotechnical challenges prevent an effective use of AI-CDSS and lead to low or discontinued use after their initial adoption [20, 30]. For example, a case study from Thai clinics implementing an AI-based DR screening tool illustrated how infrastructural inadequacies—such as poor internet connectivity and suboptimal lighting conditions—affected system performance and caused delays, ultimately reducing the tool’s value [31]. These disruptions led some of the nurses to abandon the tool and return to their old clinical routines instead. In a qualitative study on general practitioners’ perceptions of AI-enabled DR screening tools for primary care settings, the practitioners proposed several barriers and facilitators to effective usage, relating to the technology (e.g., handiness), the organization (e.g., change of established procedures), or the users themselves (e.g., attitudes towards AI) [24].

Despite these first insights, research specific to ophthalmic AI-CDSS remains limited. Most existing studies on ophthalmic AI-CDSS focus on algorithmic performance, overlooking user-centered and contextual factors that also affect the success of implementation. The few, largely survey-based studies that considered user perspectives focused on individual attitudes, neglecting external influences on adoption and use of AI-CDSS. A comprehensive investigation of the sociotechnical factors influencing AI-CDSS adoption and valuable use in ophthalmology is still missing but crucial for understanding how to implement these novel tools successfully in ophthalmic practice.

### Exploring sociotechnical influences through the NASSS framework

The NASSS framework is particularly useful for examining sociotechnical factors that influence clinicians’ (non-)adoption decisions or (dis-)continued use of new healthcare technologies [13]. Compared to other theories of technology adoption and use, the NASSS framework has a broader scope that acknowledges the context-specific and complex nature of adoption and usage of technological healthcare innovations like AI-CDSS. Specifically, this framework proposes seven domains that affect adoption of healthcare innovations and their continued use over time: *condition* (the nature of the targeted health condition), *technology* (the attributes of the technology under investigation), *value proposition* (the value the technology supposedly offers), *adopters* (the attributes of the technology’s intended users and the technology’s impact on their work), *organization* (the attributes of the implementing healthcare institution), *wider system* (the political, economic, regulatory, professional, and sociocultural context), and *embedding and adaptation over time* (the dynamic evolution of implementation and integration of the technology).

Other frameworks could have also served as the theoretical foundation for this study. For example, the Technology Acceptance Model (TAM) [32] and the Unified Theory of Acceptance and Use of Technology (UTAUT) [33] are commonly used to capture users’ evaluation of technology characteristics that influence adoption. The Sociotechnical Systems Approach by Salwei and Carayon [34] focuses on the components of successful workflow integration of AI tools into healthcare settings. The Technology, People, Organizations, and Macroenvironment framework (TPOM) [35] also enables a multi-level perspective on the implementation of healthcare technology.

We selected the NASSS framework for its specific focus on healthcare technologies and its comprehensive scope. Additionally, this framework explicitly considers temporal dynamics, acknowledging that initial adoption does not guarantee long-term use. Reviews have demonstrated the utility of the NASSS framework in synthesizing evidence on AI-CDSS adoption and use, and have recommended its application to guide data collection and interpretation in primary research [28, 36]. To our knowledge, only a single study to date has applied the NASSS framework in the context of AI-CDSS in ophthalmology, exploring stakeholder perspectives on AI for monitoring neovascular AMD treatment [16].

### Study objective

Following calls for research on user perspectives on the implementation of ophthalmic AI-CDSS [7, 26, 37], this study explores the following research question: What are ophthalmology professionals’ requirements for adoption and continued use of ophthalmic AI-CDSS in clinical practice? To identify factors influencing (non-)adoption and (dis-)continued clinical use of ophthalmic AI-CDSS by ophthalmology professionals, this study applied the NASSS framework with a particular focus on the adopter domain. To our knowledge, this is the first study that applied the NASSS framework to investigate ophthalmology professionals’ perceived or experienced sociotechnical requirements for adopting and meaningfully using AI-CDSS across multiple clinical use cases.

This study contributes to the discourse on human-centered design and implementation of AI-CDSS in healthcare. Specifically, by highlighting real-world implementation challenges and user needs, we provide actionable insights for AI-CDSS developers, policy makers, and managers of healthcare institutions aiming to implement ophthalmic AI-CDSS. The findings also inform researchers on the applicability of the NASSS framework for studying AI-CDSS adoption and use in ophthalmology.

## Methods

### Study design

This preregistered study (https://osf.io/zfw9y) followed a pragmatic qualitative research paradigm [38], which emphasizes the importance of human experiences for researching practical issues and providing actionable insights. This study received ethics approval from the local institutional review board on 27 May 2024 (approval number GEHBa-202405-V-185) and is reported according to the Standards for Reporting Qualitative Research [39] (Additional File 1). Participants provided informed consent prior to participation. To enhance transparency, an audit trail documenting key methodological decisions as well as reflexivity considerations was maintained throughout and is available on OSF (https://osf.io/yjb43).

### Data collection

We used a combination of convenience, purposive, and snowball sampling to recruit ophthalmologists and ophthalmic personnel (e.g., assistants, optometrists) from the DACH region (i.e., Germany, Austria, and Switzerland). We aimed to recruit a diverse sample of ophthalmology professionals varying in their specific roles, clinical settings, years of professional experience, as well as familiarity and experience with AI in ophthalmology. We recruited participants through a wide range of channels to increase the likelihood of broad representation across the ophthalmology profession. Specifically, we asked ophthalmology societies in the DACH region to circulate our study invitation among their members. The study was also advertised in newsletters of professional trade journals in ophthalmology, via social media and with flyers at a German ophthalmology conference. Additionally, we contacted hospitals’ ophthalmology departments and private practices using publicly available contact information and asked them to share our call for participation with their staff. Each participant received a gift card worth 20€ and the chance to win another gift card worth 50€.

Data collection took place between July and August 2024 and consisted of an online questionnaire and semi-structured interviews. The online questionnaire included demographic questions as well as questions about participants’ work in ophthalmology and their familiarity with AI in ophthalmology. Participants scheduled their interviews themselves based on their availability. The interviews were conducted by the first author via Zoom and lasted on average 24 (*SD* = 5) minutes, resulting in 8 h 53 min of audio material in total. Three interviews were conducted via phone and recorded with an external device due to technical issues or participants’ preferences.

The interview began with questions about the participants’ work as well as their familiarity and any firsthand experience with AI in ophthalmology. The main section explored sociotechnical influences on adoption decisions and requirements for a valuable use of ophthalmic AI-CDSS in practice. The questions focused particularly on the adopter domain, exploring participants’ lived experiences in their daily work, their general perceptions of AI in ophthalmology, and their expectations regarding the impact of AI-CDSS on their own work. The interview guide was tailored to account for participants’ profession (e.g., ophthalmologist vs. assistant), setting (e.g., hospital vs. private practice), position (e.g., department head vs. resident), and extent of own experience with AI-CDSS (e.g., prior use vs. no prior use). For example, whereas participants who had never used AI-CDSS for their clinical work were asked about their expectations and needs regarding the implementation of such tools, participants who had used ophthalmic AI-CDSS before were also asked about their experiences with implementing and using that tool.

The interview guide was tested with an experienced ophthalmologist and a former ophthalmic assistant, and was subsequently slightly modified. An English version of the final interview guide is provided in Additional File 2. As preregistered, we concluded data collection once the sample offered a range of different perspectives, the data provided an enriching answer to the research questions, and further data collection did not provide any more value-added insights—as evidenced by the repetition of codes and the sufficiency of the existing coding system to capture the content of later interviews. While the interview order was not intentionally randomized, we consider the risk of response bias due to interview order to be low, as we did not observe any systematic pattern between interview content and order.

### Participants

A total of *N* = 22 ophthalmology professionals (*n* = 14 ophthalmologists, *n* = 8 ophthalmic personnel) from 19 different sites across the DACH region participated in this study. Table 1 provides an overview of the sample’s characteristics. An overview of further sample characteristics at the individual level can be found in Additional File 3. Participants are referred to using labels consisting of a letter (O for ophthalmologists, A for assisting personnel) and a sequential number.

**Table 1:** Sample characteristics (*N* = 22)

Characteristics	Participants
**Country**, ***n*(%)**^**a**^	
Germany	16 (73)
Austria	3 (14)
Switzerland	4 (18)
**Age, ** ***M*** ** (** ***SD*** **)**	42.1 (13.9)
**Gender, ** ***n*** ** (%)**	
Male	9 (41)
Female	13 (59)
**Profession**, ***n*(%)**	
Ophthalmologists	
Resident	6 (27)
Attending	5 (23)
Head of Department / Medical Director	3 (14)
Ophthalmic Personnel	
Ophthalmic Assistant^b^	4 (18)
Optometrist	2 (9)
Ophthalmic Study Nurse	2 (9)
**Years of Work Experience**, ***M*(*SD*)**	16.8 (13.3)
**Facility**, ***n*(%)**	
University-Affiliated Hospital	5 (23)
Other Hospitals / Clinics	4 (18)
Group Practice	11 (50)
Optical Store / Optometry Practice	2 (9)
**AI familiarity**, ***M*(*SD*)**^**c**^	
In general	2.9 (1.1)
In ophthalmology	2.9 (1.0)
**AI-CDSS usage in daily work**, ***n*(%)**^**d**^	
Regular usage for clinical work	2 (9)
Irregular usage for clinical work	2 (9)
Unsure if regularly used tool is AI-based	4 (18)
Research on AI tools, but not used for clinical work	3 (14)
No AI usage in daily work	12 (55)

^a^One participant worked at two facilities: one in Germany and one in Switzerland

^b^One participant has worked as an ophthalmic assistant for over 30 years, and recently started to work as a business consultant for ophthalmic practices

^c^Measured on a 5-point scale ranging from 1 (not familiar at all) to 5 (very familiar)

^d^One participant conducted research on AI in ophthalmology *and* was unsure whether AI was already integrated into some devices at her facility

### Data analysis

The interviews were transcribed verbatim using the automatic transcription software whisper.ai [40] and subsequently manually corrected and de-identified. We conducted a qualitative content analysis combining deductive and inductive coding [41]. Specifically, the main code categories were deduced from the interview guide and the NASSS framework. During the subsequent inductive coding process in MAXQDA [42], these main categories were further differentiated based on the interview content. The development and application of the coding system followed an iterative approach. The first and second author independently coded a subset of six transcripts and discussed discrepancies to refine the coding system further [43]. The first author then applied the finalized code system to all transcripts, with regular discussions among all authors to ensure consistency. Additional File 4 contains the final codebook, including code definitions, frequencies, and exemplary quotes. The final codebook consists of one main code category to capture participants’ familiarity and experience with ophthalmic AI-CDSS as well as seven main code categories deduced from the NASSS framework to capture sociotechnical influences on ophthalmic AI-CDSS adoption and use. Together, inductive coding yielded 32 sublevel code categories and 20 more granular code categories nested within these. These code categories served as units of analysis. In total, 1093 coded segments emerged, ranging from 24 to 103 per interview.

## Results

### Participants’ familiarity and experience with ophthalmic AI-CDSS

To contextualize each participant’s background, participants were first asked how familiar they were with ophthalmic AI-CDSS and whether they had used such tools before. While some participants—particularly those involved in research or clinical use of ophthalmic AI tools—reported a high level of familiarity, many others described only limited exposure and knowledge in this area. Participants encountered AI in ophthalmology through various channels, including conferences, workplace exposure, and informal sources such as hearsay, with fewer participants actively seeking out information.

While almost half the sample indicated to have experience with ophthalmic AI-CDSS in their daily work at least to some extent, only four ophthalmologists were using ophthalmic AI-CDSS for clinical tasks (Table 1). Three ophthalmologists conducted research on AI in ophthalmology but did not use such tools in their own clinical practice. Four participants were unsure if the OCT devices at their facility already included AI-based features. This uncertainty reflected broader ambiguities about how to distinguish AI from traditional algorithms in CDSS (“That’s always the question, of course, what counts as AI.“ [O6]). Three participants who were not currently using ophthalmic AI-CDSS mentioned plans to adopt such tools in the near future.

Additional File 5 provides an overview of ophthalmic AI-CDSS that participants have used as part of their clinical work and how these were evaluated. While the tools were mostly regarded as useful and promising, certain shortcomings tempered the overall positive evaluation. For instance, one senior ophthalmologist noted the usefulness of the AI tool but emphasized the necessity of human intervention, particularly for atypical cases:

*So, there were actually several times when we had to override the AI and deviate from the protocol, because otherwise patient safety might have been at risk. (…) The device told us not to treat [the patient]. But it simply didn’t recognize the disease, that either a second disease had developed or the disease was presenting in an atypical location.* [O4]

### Sociotechnical influences on adoption and valuable use of ophthalmic AI-CDSS

**Fig. 1:**
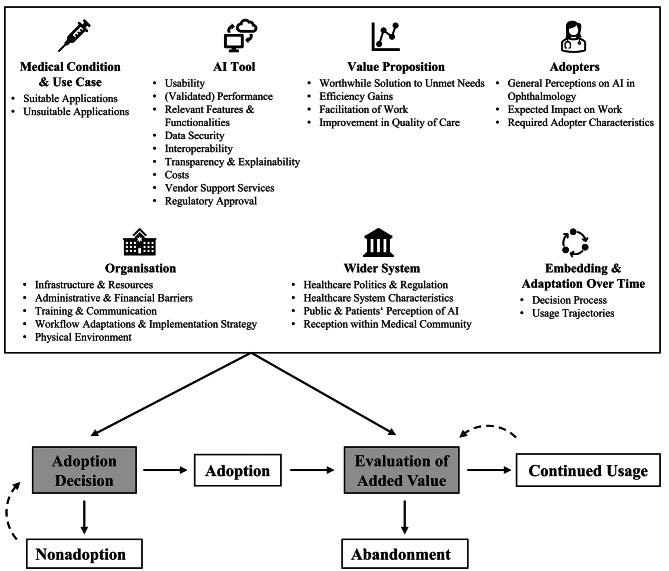
An overview of identified sociotechnical factors and visualization of their role for successful translation. Sociotechnical factors that emerged from the interviews are displayed in the textbox, grouped by NASSS domain. Critical points of decision or evaluation influenced by these sociotechnical factors are shaded in grey. Dashed arrows indicate that these decisions and evaluations may occur repeatedly over time

Figure 1 provides an overview of the 29 sub-level code categories identified—some of which further differentiated (see Codebook in Additional File 4)—that reflect influences on (non-)adoption and (dis-)continued use of ophthalmic AI-CDSS. Our findings suggest that these tools are repeatedly evaluated by weighing multiple sociotechnical factors against each other. The outcomes of these evaluations—adoption vs. nonadoption and continued use vs. abandonment—contribute to the failure or success of ophthalmic AI-CDSS within a given healthcare institution, setting the stage for broader dissemination.

In the following sections, these factors are presented according to the NASSS domains. To better align with the interview content, the NASSS domain *condition* was expanded to also capture aspects about use cases more broadly.

#### Condition and use case

Most ophthalmologists believed that AI could provide valuable support for diagnostic or treatment decisions, especially for complex cases and for eye diseases where standardized assessment is challenging.

Where I would find it helpful is in cases where even the doctors don’t agree. [O1]

Additionally, AI was considered helpful for assisting with repetitive assessments of extensive amounts of data relevant to ophthalmic decision-making.

*In ophthalmology, you’re dealing with an enormous amount of imaging material processed or recorded and produced for every patient. (…) And of course, you can imagine that after an eight-hour workday, by the time you’re seeing the 15th or 20th patient, your level of attention isn’t at its peak anymore, and subtle changes in the macula, for example, might go unnoticed.* [O8]

Specifically, AI support was deemed valuable for medical decisions relating to glaucoma, AMD, DR, and keratoconus. Many ophthalmologists supported the use of AI for screenings and referral decisions in primary eye care, particularly in underserved areas. Less frequently mentioned applications included predictions of disease progression, research, and triage. In contrast, participants considered use cases requiring extensive patient interaction, such as neuro-ophthalmology or post-surgical examinations, as unsuitable applications. Similarly, ophthalmic personnel generally described ophthalmic AI as more suitable for image interpretation and screening rather than patient-facing tasks like anamnesis.

#### AI tool

The tool’s usability was paramount for participants’ adoption decisions and for deriving (or expecting to derive) added value post-adoption. One aspect of usability was that the tool should be easy to learn and to use, so that its value can be derived quickly after implementation.

*Given the staff shortages, the question is how steep the learning curve is. If (…) it turns out to be more complicated than expected, then it becomes difficult because personnel resources are already so limited.* [O11]

In line with this notion, participants emphasized the importance of an intuitive interface without unnecessary or distracting features:

*The analysis also needs to be clear, not have too many different functions (…) Especially with these OCT examinations, there are already so many different features. Everyone looks at them a bit differently somehow. So it’s not really standardized because there are so many features.* [O10]

Moreover, participants stressed that using the tool should not slow down workflows, for example due to time-consuming data processing or manual steps and clicks.

*We have enough programs where you need already ten clicks to get to where you want to be. (…) Ideally, I have the dataset and press a single button.* [O7]

Other aspects of usability included delegability to assistants and patient friendliness.

Besides usability, performance was another key factor for adoption decisions and effective post-adoption usage, with participants noting the need for reliable software that delivers high-quality results. Additionally, whether the tool’s accuracy was also validated through clinical studies was an important adoption consideration.

Specific tool features considered useful included the ability to learn from user feedback and to integrate multiple data inputs from different devices or examinations.

*If you use AI tools for a specific diagnosis, the AI of course has to take multiple devices into account. (…) For example, in glaucoma, it’s not just the OCT image or imaging alone that matters, but of course, it would also be helpful to have perimetry included, or intraocular pressure, for example. So something along the lines of combining that information.* [A5]

The participants also valued functionalities such as flagging abnormalities, suggesting differential diagnoses besides the most likely diagnosis, suggesting different treatment alternatives, and providing visualizations. The ability to automate tasks (e.g., setting up OCT measurements) and to detect human errors (e.g., incorrect data entry) were also considered valuable features, particularly for the assistants’ workflows. Another desired feature was the possibility to customize the tool to accommodate different clinical settings and users.

Other, less frequently mentioned tool-related considerations included data security, interoperability, transparency/explainability, and costs. Regulatory approval was identified as a prerequisite for adoption, while vendor support for troubleshooting was seen as crucial for ensuring effective use after implementation.

*That perhaps the program itself includes a help function, when problems come up. There should definitely be some kind of direct contact (…), an emergency number or an emergency support service.* [O13]

#### Value proposition

Overall, participants mentioned three main dimensions on which ophthalmic AI-CDSS should deliver value: efficiency gains (primarily through time savings), facilitation of work (e.g., by automating repetitive tasks), and improvement in quality of care (e.g., by detecting diseases more reliably).

*If—because of automated analyses—physicians spent less time examining each individual cross-sectional image and comparing it with others, that would save time. And this time could then be used, for example, to explain things to the patient in more detail or to perform additional treatments. As for quality, if unnecessary injections or treatments could be avoided as a result, that would please both the patients and the hospital, of course.* [O4]

Another participant emphasized the potential of AI-CDSS to provide value especially in screening workflows by dividing tasks between physicians and AI more effectively:

*We won‘t waste our time with screening. Instead, we need to properly treat screened patients. We shouldn’t focus the physician’s extremely expensive working time on tasks that a machine can do. I would rather train people to focus on high-quality tasks later on than burn them out by having them look at normal findings.* [O2]

Moreover, half of the participants emphasized that they would only adopt AI-CDSS if these provided a worthwhile solution to unmet needs. One of the most prominent reasons why participants have not (yet) adopted an ophthalmic AI tool was skepticism about the merit of currently available tools (“There’s no product on the market that would offer us enough added value that getting it is worth it.“ [O4]). Participants highlighted the need for a favorable cost-benefit ratio. A few ophthalmologists also noted that existing workflows already met their needs, making AI-CDSS adoption unnecessary unless it significantly improved outcomes or streamlined processes.

*Because you don’t really need these programs. (…) When I take an image and interpret it, I don’t need AI in addition. (…) Of course, the AI could look at it beforehand based on many other images. You input it and see if there’s something there or not. That’s true—but as a doctor, I see that too.* [O11]

Still, participants with prior AI-CDSS experience described how the tool facilitated decision-making and increased their confidence.

*I noticed that AI sometimes detects things that weren’t visible to me with the naked eye or didn’t seem significant because there’s simply so much data at once. And it very quickly identified which values and things were important, and provided quite helpful support.* [O4]

#### Adopters

Within the adopter domain, we explored (1) participants’ general perceptions of AI in ophthalmology, (2) their context-specific expectations about how AI-CDSS might affect their own work, and (3) any own personal requirements they considered necessary to derive added value from AI-CDSS in clinical practice.

##### General perceptions on AI in ophthalmology

The general perception of ophthalmic AI among participants was largely positive, with most expressing curiosity, openness, and recognition of its potential given the growing need for ophthalmic care (“I see a huge benefit. That we need support especially in these times, where we have a shortage of skilled staff, little time, fewer people, more patients, more illnesses, and more treatment options.” [O6]). For example, 15 out 22 participants stressed that ophthalmology offers great potential for the use of AI due to the discipline’s technological affinity and image-based diagnostics (“We generate an incredible amount of data, an incredible number of imaging tools, etc. So in that sense, ophthalmology is almost predestined for this.” [O7]).

Participants viewed AI in ophthalmology as a rapidly evolving but still developing technology. Even though available AI models were largely perceived as not yet fully mature (“I think it’s still a bit too underdeveloped, because it’s always designed for just one device.” [A5]), there was a strong consensus that AI will play an increasingly important role in ophthalmology, with some participants expecting AI-driven tools to become standard in clinical workflows in response to demographic shifts and workforce shortages.

Although most participants expressed optimism about a future with greater AI involvement, they still perceived a handful of general risks and limitations of AI, including concerns about accuracy, transferability to different settings, commercial interests, and overreliance on technology.

*As is the case in research, it ultimately comes down to generating revenue, financial gain. For example, there are many studies and only the ones that serve the company’s interests end up being made public.* [A8]

Interestingly, nearly one-third of participants referred to large language models, such as ChatGPT, when discussing ophthalmic AI-CDSS, suggesting that these also influenced their perceptions of AI in ophthalmology or served as reference point for both positive and negative comparisons.

*That, depending on how it’s programmed, there can also be errors. For example, with ChatGPT, I once had to give a presentation and asked it for help, and it lied. It gave me false information.* [A8]

##### Expected impact on work

Participants were asked in what ways they expected the introduction of AI-CDSS to change various aspects of their everyday work. Five categories emerged.

##### Challenges and risks

Some participants anticipated implementation challenges, such as workflow disruptions and difficulties with ensuring data security during technical setup.

*For me personally, [I would] definitely [expect problems or challenges related to] the IT side—making sure it’s compatible, because that’s something I find overwhelming. (…) And also security and data protection, those are definitely things that need to be looked at closely. (…) So those are psychological barriers that need to be overcome.* [A8]

Nevertheless, some participants perceived little difference between the introduction of ophthalmic AI-CDSS and other new technologies, both entailing comparable implementation challenges and adaptation processes. For them, ophthalmic AI-CDSS is part of the ongoing evolution of medical practice:

*I do think it’s a challenge, but not because it’s AI, but because everything new is always a challenge. I don’t think AI is much more than just another tool being introduced here.* [A1]

In contrast, some participants expected the AI component to trigger resistance among colleagues and patients or to increase the risk of negligence due to overreliance on AI output. At the same time, some participants explicitly stated that some of the perceived risks could be minimized by proper safeguards and do not necessarily deter adoption (“I think as long as the data stays within the practice, like with our current devices, I don’t see any major risk.” [O10]).

##### Quality of care

The majority of participants expected the introduction of ophthalmic AI-CDSS to improve quality of care. Specifically, AI-CDSS were expected to enhance diagnostic accuracy and standardize decision-making by supporting clinicians in analyzing large amounts of data, identifying subtle changes, alerting clinicians to easily missed abnormalities, and reducing subjectivity in treatment decisions.

*The ultimate goal, of course, is for the patient to be treated correctly and appropriately (…). And I can achieve that by getting the most out of all my diagnostic images—and not, for example, overlooking something. (…) I’m not saying that all patients are being treated incorrectly, but AI can undoubtedly make a significant improvement.* [O7]

##### Working conditions

The majority expected ophthalmic AI-CDSS to facilitate their work and increase efficiency, for example, by automating routine tasks, accelerating image analysis, and enhancing decision-making through more structured and comprehensive information. However, some participants cautioned that these benefits would only materialize if the tool met certain conditions (e.g., high quality, reliability, relevant features) and was used appropriately.

*I think [my work] would change in the sense that, if the tool works reliably, you would of course still review everything and bring the findings together, but it could definitely provide some relief.* [O14]

Although most participants viewed AI as a tool that would optimize workflows and decision-making in their everyday work, they did not necessarily expect their workload to decrease. This was mainly due to the assumption that efficiency gains would simply translate into higher patient volumes or that the introduction of a new tool would cause additional work—which participants who had used ophthalmic AI-CDSS confirmed:

*Of course, in the initial phase it meant extra work, because you had to familiarize yourself with the product, deal with technical issues in the background, and adjust the workflows.* [O4]

##### Tasks and professional identity

Most ophthalmologists agreed that AI-CDSS would not affect their professional identity, even though some tasks and workflows might change. Half of the participants expressed that they viewed ophthalmic AI only as an assistive, optional tool rather than a replacement for human expertise, therefore not affecting the core of their professional identity (“If I don’t want to use it, or don’t want to use it with a particular patient, I don’t have to. It’s just an assistance.“ [A5]).

Moreover, some ophthalmologists anticipated their role shifting to an AI supervisor, ensuring accuracy rather than generating diagnoses from scratch—a task for which they still considered their expertise essential:

*That you still keep an overview of the measurements yourself and maintain a general understanding, so you can recognize incorrect measurements or things like that. (…) I think the work itself will simply change a bit, but the role should basically stay the same.* [O13]

While most ophthalmic assistants did not expect the introduction of AI-CDSS to affect their professional identity, at least not in the near future, one assistant was concerned that the introduction of AI-CDSS might reduce the need for assistants like her. In contrast, some ophthalmologists believed the introduction of AI-CDSS could increase task delegation, leading to a greater role for ophthalmic personnel in clinical processes.

##### Patient relationships

The final category of expected impact concerned participants’ relationships with their patients. Participants’ expectations were mixed: Some feared reduced personal interactions, while others believed efficiency gains could allow for more meaningful engagement. Opinions diverged on whether AI involvement would strengthen trust in diagnoses or foster skepticism.

*If it’s communicated directly, it might [have an impact] because, in general, AI is a topic on which opinions in society are highly split.* [A5]

*Depending on how I explain it, it could actually provide patients with an added sense of security because I, as a doctor, am only human, and have an additional machine on top.* [O5]

Three ophthalmologists who had used AI-CDSS in clinical practice noted that patients remained unaware of its use, as the systems operated in the background. Among other participants, opinions on the necessity and extent of disclosure varied, emphasizing the challenge of balancing informational needs, patient rights, and consultation efficiency.

##### Required adopter characteristics

Participants were also asked about any personal requirements they felt they would need to fulfill in order to derive added value from AI-CDSS. Most participants agreed on the importance of their own adoption readiness, which they described as openness towards the new technology, willingness to learn, interest, and flexibility. Adequate usage and knowledge also emerged as critical adopter-related requirements for valuable usage: Alongside sufficient technical proficiency and ophthalmic expertise, participants emphasized the importance of using the tool as it is intended, understanding its operations, interpreting its output accurately, recognizing limitations, and identifying potential errors. This also included a balanced level of trust in the tool (i.e., neither blind trust nor complete distrust).

*That all staff members, from all professional groups who come into contact with it, know how to use the technology properly, and (…) that the capabilities and limitations of the device can be adequately assessed.* [O14]

### Organization

Participants identified several infrastructural requirements—technology, workforce, and support resources—for both adoption and effective usage of AI-CDSS in clinical practice. Technical requirements included high-performance computers, compatible devices, up-to-date software, digital workflows, and reliable internet connectivity.

*Well, for example, we don‘t have the latest software for our OCT devices or the fundus cameras, and currently at the university hospital, we haven’t had internet connection since October [eight months]. So, I do engage with this topic, but at the moment we have no way to actually use them.* [O3]

The importance of technical infrastructure for effective use of AI-CDSS was also highlighted by an ophthalmologist’s negative experience:

*One issue was that the fundus camera was XP-based and couldn’t be updated, which meant the images had to be transferred manually and then uploaded. That was the most frustrating part.* [O2]

Participants regarded the availability of sufficient qualified and accepting staff as an equally important requirement. One ophthalmologist suggested that attitudes among the workforce might be even more critical than technical infrastructure:

*When you think in terms of software-based AI tools, it’s not so much a major hardware change, it’s actually more about the mindset.* [O8]

Several ophthalmologists emphasized the need for immediate IT support in case of technical issues, as prolonged system downtimes would severely disrupt clinical workflows:

*In the end, there must be someone who can operate the system, who knows how to troubleshoot issues, and who can resolve them immediately. (…) Because the workflows in an eye clinic or ophthalmology practice have such a high patient throughput that if a program fails, it becomes a major problem if it isn’t fixed within an hour.* [O12]

To accommodate such new demands after tool implementation, participants deemed corresponding financial expenses necessary:

*It’s also not feasible for staff to keep using the same number of monitors as before because they can’t constantly switch between windows on the same screen. So surely, an IT upgrade at each workstation is necessary. (…) From my experience with various clinics, IT departments are already stretched to their limits. (…) And if this new technology is introduced and the IT department is asked to take it on as well, that would only be possible with additional staff.* [O7]

However, administrative and financial barriers, including complex data processing requirements and reluctant higher-level decision-makers, hindered adoption.

*We’d be very happy to have such a tool starting tomorrow. There really aren’t any other barriers. It’s just that it costs money to acquire something like that and that you have to make sure to comply with all the regulations.* [O7]

Besides infrastructure and financial resources, comprehensive training and clear communication also emerged as important organizational requirements: Almost all participants emphasized the need for user group-specific onboarding that addresses the tool’s intended use and workflow adjustments. Sufficient time for familiarization and clear communication were deemed essential for addressing staff needs.

Nearly half of the participants further emphasized the need for careful planning to ensure that the AI tool aligns with overarching institutional strategies and integrates smoothly into existing workflows. This included analyzing potential challenges and necessary workflow adaptations in advance. Specifically, some participants considered a human-in-the-loop approach and clear guidelines for patient communication essential for new workflows. Participants with own AI-CDSS experience, who witnessed changes in workflows and tasks following AI adoption, highlighted the need for inter-professional collaboration among all affected personnel to design and document new workflows:

*I think the fundamental problem was that the workflow changed slightly, and any workflow changes within a clinic mean you have to bring everyone on board—from the nurses to the assistant who performs the OCT. (…) And especially with the number of staff we have, ensuring that everyone really understands how things work in the new workflow, I think that’s the biggest challenge, which sometimes leads to difficulties at the beginning.* [O12]

Finally, some participants considered short walking distances, accessibility from multiple computers, and sufficient space for new devices important for effective usage of newly implemented AI-CDSS.

### Wider system

Aspects about the wider political or societal system also influenced participants’ adoption decisions. For example, regulations for AI as medical devices were seen as both beneficial and challenging: While ensuring the approval of only safe and accurate AI tools, they also complicate the implementation process within participants’ own institutions:

*When you use such a cloud-based tool, are you complying with the regulations? As a clinic or clinician, you don’t want to risk anything by uploading your patient data into an AI tool.* [O8]

Also, the prospect of external cost coverage played a role since additional expenses need to be financially supported (“Acquiring all of this is going to cost money. Politics has to support this somehow. Otherwise, no one will be motivated to do so.“ [O1]).

Participants also considered healthcare system characteristics: The limited telemedicine integration and insufficient digital infrastructure hindered adoption, whereas workforce shortages increased adoption intentions. AI acceptance among the general public and patients were also reported to encourage adoption. Moreover, three participants said that their adoption decisions would be influenced by the medical community’s reception of AI, particularly through clinical guidelines or shared experiences from other institutions:

*It would of course be desirable to have a reference clinic that already has experience with this tool, where you could see how it’s been implemented there because that would make the introduction easier and then you also know that it delivers what it promises.* [O4]

Some participants also lamented resistance within the medical community, often driven by fear of replacement or misconceptions about AI:

*Especially the older generations, they’re not as tech-savvy and believe certain myths or falsehoods. And that’s actually a major barrier to why AI applications aren’t that widely used in medicine yet.* [O8]

### Embedding and adaptations over time

The decision-making process for adopting ophthalmic AI-CDSS varied across institutions and involved multiple stakeholders. Almost a third of participants expressed a preference for exploring, comparing and testing available tools before making an adoption decision. At least at larger facilities, the final decision on whether to acquire an AI tool was (or would be) typically made at higher administrative levels, involving department heads, IT departments, and senior ophthalmologists. For the selection of a specific tool, input from the physicians and, in some cases, assistants was (or would be) considered. Clinical directors and lead physicians were reported to play a key role in evaluating AI tools and presenting them to colleagues:

*If the prerequisites are right, then we would present the product to the larger team. Because in the end, the doctors are the ones who have to work with it and if they perceive added value, they’re usually very willing to do so. So the first step is to see what the product can do, and then we could demonstrate what it can do.* [O4]

Multiple sociotechnical factors that argue for or against the introduction of AI-CDSS are weighed against each other (“You really have to do a cost-benefit calculation; it has to check out.“ [O2]). Participants emphasized, however, that nonadoption decisions may be later reversed. For example, some interviewees noted that currently available tools are not yet sufficiently developed but could be considered for adoption in the future.

Regarding the dynamics of post-adoption usage, participants anticipated an initial familiarization period, during which they would need to adapt to new workflows, learn how to delegate tasks to AI, and continuously reevaluate and refine processes (“And then maybe also meet regularly over time and evaluate whether everything is working the way it should.“ [O3]). Those with AI-CDSS experience reported such adaptation processes took time:

*The processing of the OCT data had changed, and it was still being handled according to the old system instead of the new one. That was at the beginning; we had to retrain [the new workflow] once in a while.* [O12]

Participants also described that the tool, once implemented, would be evaluated for its added value. If the burden, however, outweighed its benefits, some participants predicted they would abandon the tool (“But if the AI doesn’t manage [to save resources] and even ends up creating more work for me, (…) then it’ll be dismissed fairly quickly.“ [O6]). In line with this view, one of the ophthalmologists switched to another AI tool since the first tool created more work than value:

*The biggest downside is that you have to upload the data, and that’s an extra step. That’s something that ultimately bothered me and led me to now use another tool that generates and syncs the data automatically.* [O2]

Almost a third of participants expected a gradual trust building process, emphasizing the need for personal experience and validation over time. Many indicated they would initially run AI analyses in parallel with their own assessments to verify accuracy. Some viewed AI-CDSS as similar to a junior colleague whose decisions require close scrutiny before gaining trust. Over time, as AI-CDSS prove reliable and beneficial, participants predicted that skepticism would likely decrease, leading to routine use.

*Then you would try it out yourself and see whether it aligns with your own assessments, or let it weigh in on simpler cases and double-check yourself, so to say. And if that works out, I think that would reduce my concerns.* [O9]

With prolonged AI use, however, participants feared the risk of overreliance, where clinicians might increasingly accept AI-generated outputs without critical evaluation, potentially overlooking errors and diminishing their own diagnostic skills.

*Of course it could happen that doctors, who currently deal a lot with the images themselves and basically examine them thoroughly themselves, might stop looking at them as carefully in the future (…) and instead focus only on the text the AI provides (…) because of course you might trust the AI tool more than your own judgment, or because it’s also more convenient.* [O7]

## Discussion

This study explored sociotechnical factors that influence ophthalmology professionals’ decision to adopt ophthalmic AI-CDSS and their ability to derive added value from these tools once implemented in clinical practice. The findings offer new insights into how AI-CDSS can be successfully translated into ophthalmic practice—a prerequisite for realizing their potential to enhance patient care and working conditions in ophthalmology. Three major findings arose: First, most participants were open to these tools, especially as a means to complement their own expertise and skills, facilitate their work, and improve patient care. Second, ophthalmic AI-CDSS undergo repeated sociotechnical evaluations of their expected and experienced value relative to their costs, both pre- and post-adoption. Third, many of the identified sociotechnical factors and challenges are also relevant to traditional healthcare technologies and other medical disciplines, although some ophthalmology- and AI-specific considerations emerged as well.

**Fig. 2:**
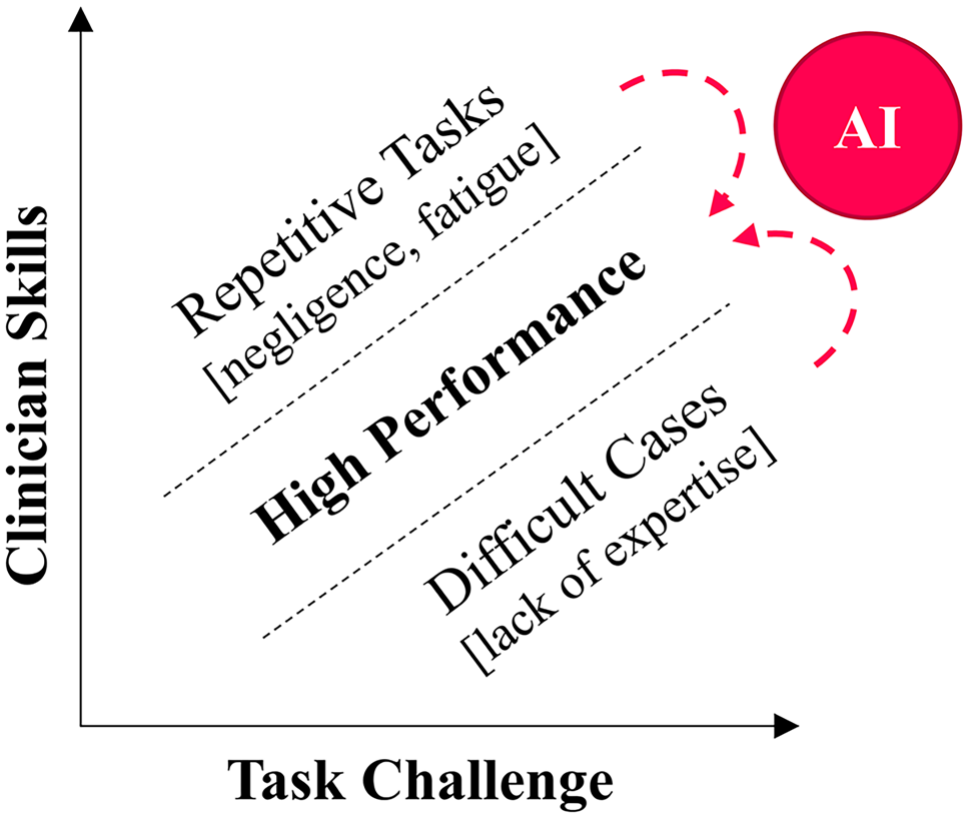
AI as a means to balance task requirements and clinician skills

Participants broadly acknowledged the need for support amid the increasing workload and growing complexity of clinical decision-making. Like previous accounts that position ophthalmology as a particularly promising field for AI applications [7], many participants saw potential in ophthalmic AI-CDSS—especially as these mature—to support image-based decision-making. Two main areas emerged where AI-CDSS support was considered valuable: First, assistance with complex cases to complement own capabilities or provide a second opinion, and second, assistance with easy, but repetitive assessments to prevent oversights due to fatigue or inattention (Fig. 2). This finding points to a role of AI-CDSS in helping to balance the relationship between the task’s challenge and the clinician’s skills—a notion that aligns with the concepts of *human-AI synergy* or *hybrid intelligence* (i.e., increasing task performance by combining the respective strengths of humans and AI) [44, 45].

Even though the participants echoed some concerns raised in prior studies—such as data security, accuracy, and overreliance [23–25]—most were open to incorporating AI-CDSS into their clinical work given that some risks could be mitigated with appropriate safeguards. While prior studies reported mixed perceptions among ophthalmology professionals regarding the threat of AI to their autonomy or professional identity [25, 46, 47], the comments made by the professionals in this study offer a different perspective: Participants described how ophthalmic AI-CDSS may complement rather than replace them, potentially opening new perspectives toward cautious acceptance.

However, the findings also indicate that openness to adopting AI-CDSS and favorable perceptions of these tools do not necessarily translate into actual adoption. Both in healthcare and other domains, the perceived ease of use and perceived usefulness of AI tools were highlighted as important predictors of use intentions [43–49]. Our findings suggest these are necessary but not sufficient requirements for adoption. A range of sociotechnical factors beyond individual attitudes and perceptions also emerged as important influences, such as workplace and healthcare system characteristics. Some participants, for example, were willing to adopt ophthalmic AI-CDSS but were deterred by external factors, such as the lack of resources and support at their facility, societal AI acceptance, or digitalization within the healthcare system.

The findings further showed that sociotechnical factors are also relevant post-adoption, namely for deriving added value from newly implemented AI-CDSS. In line with reviews finding that newly introduced AI-CDSS do not always yield clinical benefits [20, 21, 50], participants with and without AI-CDSS experience identified a range of requirements for effective use of AI-CDSS in their daily work, such as good training and support structures. Many of these challenges and requirements mirror those reported by previous studies within other medical disciplines [28, 51, 52], highlighting cross-disciplinary requirements such as usability, training, technical support, workflow alignment, and adequate infrastructure.

Nevertheless, three challenges emerged that appear particularly pronounced in ophthalmology. First, ophthalmic decision-making usually integrates multiple data sources (e.g., different imaging modalities combined with clinical examinations). Consequently, the currently available AI-CDSS tools’ reliance on single-modality input was perceived to limit their trustworthiness and practical usefulness. Second, participants indicated that the typically older patient population may be less receptive to AI involvement in their care, prompting participants to reflect on how to best communicate the use and results of AI-CDSS to these patients—or whether to use AI-CDSS during their consultations at all. Third, some participants stated that diagnostic and treatment decisions within ophthalmology often involve considerable clinical disagreement, making them cautious about the ground truth used to train AI models. While some saw AI as a possibility to increase standardization, others feared a false sense of objectivity.

Both in the literature and this study, there appears to be a substantial overlap between the implementation challenges of AI-CDSS and digital tools not based on AI [36, 53, 54]. For example, participants noted that many of the anticipated or experienced implementation challenges of AI-CDSS resemble those encountered with previously implemented tools, including modifications to existing workflows, financial constraints, and training demands. This overlap may be particularly pronounced in a technology-driven field such as ophthalmology, where (digital) technology is already integral to clinical practice, compared to less technology-intensive fields (e.g., mental healthcare). Consequently, when integrating AI-CDSS, ophthalmology professionals and facilities may be well-positioned to leverage their prior experiences with implementing digital tools.

Nevertheless, a few AI-specific challenges also emerged, particularly concerning the psychological appraisal of AI-CDSS. For example, participants described that the need to navigate novel regulatory, data processing, or patient disclosure requirements created uncertainty and raised the psychological threshold for adoption. Many also reported or anticipated skepticism—or outright resistance—toward AI involvement among their colleagues, hospital management, the wider medical community, or patients. Some participants acknowledged that trusting a technology they did not fully understand was not just a hurdle for others but also for themselves. Contrasting the cautious perspectives, some participants expressed very high expectations for care improvement and efficiency gains, despite the still limited evidence supporting these outcomes [21]. Compared to previous healthcare technologies that attracted less public attention, (ophthalmic) AI-CDSS may carry a higher risk of disillusionment if these expectations are not fully met. These considerations suggest that within the NASSS framework, the adopter domain is especially critical for successful AI-CDSS integration. Their psychological appraisal of AI-CDSS may be what sets these tools apart from traditional healthcare technologies. However, further research is needed to better understand how sociotechnical requirements differ between AI and non-AI technologies in healthcare.

This study also complements existing research by shedding light on the temporal dynamics of (ophthalmic) AI-CDSS adoption and usage—a NASSS domain that has often been neglected in prior research on CDSS implementation [53, 55]. Participants described a multi-phase, sometimes recursive process: from initial explorations and testing through institutional decision processes and strategic planning to implementation, onboarding, and early troubleshooting. Finally, gradual integration into clinical routines was expected to include a familiarization phase and dynamic trust building process, shaped by repeated evaluations of system accuracy and added value against financial and psychological costs. Notably, when considering adoption, participants already considered factors that only become relevant *after* adoption (e.g., onboarding and IT support), highlighting the interplay between expectations of future challenges and present decision-making. Future research could investigate which NASSS domains and sociotechnical factors are most influential for different obstacles that AI-CDSS face on their path to widespread use (e.g., adoption, post-adoption use, wider dissemination, sustainability). Such insights could inform the design of phase-specific strategies to help overcome the AI chasm in healthcare.

### Theoretical contributions

This study provides a structured, theory-informed exploration of sociotechnical factors shaping adoption and use of ophthalmic AI-CDSS. Whereas previous work within ophthalmology has often examined these factors in isolation—focusing for example only on technical performance or on users’ perceived benefits and concerns—our application of a comprehensive framework allowed us to contextualize such factors within the broader sociotechnical environment [17, 28, 56].

A recent scoping review highlighted that many studies on the implementation of AI in healthcare either lacked theoretical grounding or relied on generic frameworks not tailored to the intricacies of healthcare or AI [51]. While the NASSS framework was designed for the healthcare context, it does not specifically target AI. Recent studies exploring AI implementation using the NASSS framework have reached mixed conclusions about whether it requires adaptation for AI-specific challenges [55, 57]. Our deductive and inductive coding process found that the NASSS framework adequately fit the interview content: All seven NASSS domains were represented, and no additional higher-level categories were needed to also record AI-related factors. Instead, AI-related aspects—such as trust, literacy, and regulation—were adequately captured within the existing domains.

Nevertheless, in the context of ophthalmic AI-CDSS, it proved useful to broaden the NASSS domain *condition* to include aspects beyond specific eye diseases. As ophthalmic AI-CDSS encompass a variety of tools designed for different applications, participants reflected not just on the utility of AI-CDSS for specific eye diseases, but also on the suitability of use cases more broadly, including the clinical task (e.g., screening) and clinical setting (e.g., primary eye care). Additionally, for AI-based systems, this domain may benefit from considering input modality as well (i.e., the type of image analyzed). For example, instead of targeting only one eye disease, some ophthalmic AI-CDSS analyze a specific image modality (e.g., OCT) to generate insights applicable to multiple conditions. A broader scope of *condition* may thus provide a more comprehensive understanding of users’ considerations relating to this domain.

Moreover, this study contributes to the existing literature by applying the NASSS framework not only to identify barriers and facilitators to adoption—an area that has dominated prior research—but also to explore the post-adoption requirements that influence the continued use of AI tools in clinical practice. Our findings highlight that sociotechnical factors do not cease to matter after implementation. Rather, they influence whether ophthalmic AI-CDSS deliver added value in practice and are ultimately retained by clinicians and healthcare institutions—a prerequisite (alongside initial adoption) for crossing the AI chasm.

### Practical implications

Despite calls from the American Academy of Ophthalmology for clearer guidance on implementing AI in ophthalmology [37], the field still lacks consensus on best practices. This study addresses this gap by providing developers and implementers with actionable insights. For AI developers, the findings offer valuable information about desired functionalities and characteristics of ophthalmic AI-CDSS, such as minimal steps for operation and vendor support services. For AI implementers, the study highlights end users’ needs and expectations, shedding light on specific implementation challenges and desired support structures.

For example, participants identified the development of adequate training units to accompany the introduction of ophthalmic AI-CDSS as a future challenge. They indicated that such training should not only explain the AI tool’s appropriate use and new workflows but also facilitate an understanding of limitations and appropriate levels of trust. Additionally, training should consider the users’ needs, expectations, and concerns. Designing effective training therefore requires the collaboration between AI developers, researchers, and ophthalmology professionals.

The findings also carry policy implications. Practical guidelines for data processing and patient disclosure as well as clear, accessible communication of clinicians’ and healthcare institutions’ obligations are needed to lower the psychological threshold to AI-CDSS adoption and use in ophthalmology. The mixed opinions on patient disclosure in this sample further suggest the need for multi-stakeholder discussions on how to balance patients’ informational rights while ensuring practicability within everyday clinical routines [58, 59].

### Limitations

Several limitations should be acknowledged. First, the participants who volunteered for the study might have been more interested in AI than the general ophthalmic workforce, potentially skewing responses toward more positive views. Although we advertised our study to a broad range of ophthalmology professionals, some of the recruitment channels (e.g., newsletters, conferences) likely reached especially those interested in current developments in their discipline. Own experience with AI-CDSS might have also motivated professionals to take part, whereas those with less exposure or knowledge might have been more hesitant to participate in an interview study. We attempted to mitigate such biases through purposive sampling and by highlighting in our call for participation that no prior AI-CDSS experience was needed. Overall, the resulting sample included both progressive and cautious perspectives. However, due to the possibility of self-selection and the limited sample size, we do not claim representativeness of all ophthalmology professionals in the DACH region.

Second, the anticipated or experienced challenges of AI-CDSS implementation likely do not provide a complete account of potential issues. As prior work noted, unanticipated sociotechnical problems often arise during implementation [60]. Also, individual experiences recorded in this study may not generalize to other AI-CDSS tools or clinical settings. Similarly, generalizability beyond the DACH region is limited. Countries in this region share similar healthcare infrastructures and regulatory environments, which may not reflect conditions elsewhere. In particular, the potential of AI-assisted screening in resource-limited settings (e.g., in developing countries) underscores the need to investigate implementation across diverse healthcare systems. In sum, more research is needed that evaluates the real-world implementation and practical value of ophthalmic AI-CDSS in diverse healthcare contexts.

Third, in order to accommodate for the busy schedules of ophthalmology professionals, the interviews were intended to last around 25 to 30 min. Longer interviews would likely have allowed for more in-depth discussions. However, we decided against a mandatory longer format, as this could have systematically excluded professionals with limited availability, such as parents or head of departments. In practice, the semi-structured interview guide was applied flexibly, tailored to participants’ availability and prior responses. Some participants, for example, were only able to schedule the interview during work breaks. Other interviews were naturally shorter when participants were less familiar with AI-CDSS, gave brief responses, or preemptively addressed later topics of the interview guide.

Finally, the study focused on two stakeholder groups: ophthalmologists and supporting personnel. However, successful implementation of AI-CDSS involves multiple stakeholder groups, including patients, carers and members of the general public, AI developers, healthcare managers, regulatory bodies, and policymakers [61]. Although some participants held leadership positions and were able to provide insights into higher-level administrative challenges, input from other stakeholder groups is especially important to shed light on organizational and system-level requirements for AI-CDSS adoption and effective use. Notably, multi-stakeholder perspectives remain underrepresented in the current literature [61]. Future studies could expand the stakeholder base to better understand broader implementation dynamics, including long-term sustainability and scaling of AI in ophthalmology [16, 62].

## Conclusion

This study offers a theory-informed overview of sociotechnical factors that influence ophthalmology professionals’ adoption of AI-CDSS and their ability to derive added value from these tools in clinical practice. The findings highlight the importance of examining these factors not in isolation, but through a comprehensive, systems-oriented lens. While many challenges associated with the implementation of AI-CDSS mirror those of other healthcare technologies, the findings suggest that an important difference lies in the users’ psychological appraisal of AI-CDSS. The findings support the theoretical applicability of the NASSS framework in the context of AI-CDSS and provide actionable guidance for AI developers and implementers. Future research should explore the perspectives of other key stakeholders and identify challenges to the scale-up, spread, and sustainability of ophthalmic AI-CDSS to gain a more complete understanding of how to overcome the AI chasm in ophthalmology. Ultimately, this study advances our understanding of how to bridge the gap between AI-CDSS development and their valuable use in ophthalmic practice, helping to realize their potential for real clinical impact.

## Supplementary Information

Below is the link to the electronic supplementary material.

Additional File 1: Standards for Reporting Qualitative Research (SRQR): 21-item checklist.

Additional File 2: English version of the semi-structured interview guide.

Additional File 3: Additional table with sample characteristics at the individual participant level.

Additional File 4: Codebook including code definitions, frequencies, and exemplary quotes.

Additional File 5: Additional table with a description and evaluation of the AI-CDSS that participants had used for their clinical work.

## Data Availability

The interview guide and the codebook are presented in Additional File 2 and Additional File 4. The interview transcripts are not publicly available for data protection reasons but are available from the corresponding author on reasonable request.

## Footnotes

1 A PubMed search [May 2025] on ophthalmic AI yielded over 1800 entries, with an exponential increase over the years. Search term: (ophthalm*[Title] OR OCT[Title] OR fundus[Title) OR AMD[Title] OR diabetic retinopathy [Title] OR glaucoma[Title] OR geographic atrophy[Title] OR retinopathy of prematurity[Title] OR cataract[Title]) AND (artificial intelligence[Title] OR AI[Title] OR machine learning[Title] OR deep learning[Title] OR DL[Title]).

## Abbreviations

AI: Artificial intelligence
AI-CDSS: Artificial intelligence-enabled clinical decision support systems
AMD: Age-related macular degeneration
DR: Diabetic retinopathy
NASSS: Nonadoption, Abandonment, Scale-Up, Spread, and Sustainability
OCT: Optical coherence tomography
