# Supplementary material for: Sociotechnical influences on the adoption and use of AI-enabled clinical decision support systems in ophthalmology: a theory-based interview study
Source: BMC Health Serv Res. 2025 Oct 22;25:1398. doi: 10.1186/s12913-025-13620-w (PMC12542331; doi:10.1186/s12913-025-13620-w)
Supplement: Supplementary file 1 — Additional File 1: Standards for Reporting Qualitative Research (SRQR): 21-item checklist. [file 12913_2025_13620_MOESM1_ESM.docx]

**Standards for Reporting Qualitative Research (SRQR)**

Based on O’Brien BC, Harris IB, Beckman TJ, Reed DA, Cook DA. Standards for Reporting Qualitative Research: A Synthesis of Recommendations. Acad Med 2014 Sep;89(9):1245–1251. doi: 10.1097/ACM.0000000000000388.

| **No.** | **Topic** | **Item** | **Location in Manuscript (section)** |
| --- | --- | --- | --- |
| S1 | Title | Title identifies the study as qualitative and mentions the approach or data collection methods | Title |
| S2 | Abstract | Abstract summarizes background, purpose, methods, results, and conclusions | Abstract |
| S3 | Problem formulation | Description and significance of the problem; review of literature and theory | Background |
| S4 | Purpose or research question | States the research objectives or questions | Background |
| S5 | Qualitative approach and research paradigm | Specifies qualitative approach and paradigm | Methods |
| S6 | Researcher characteristics and reflexivity | Describes researcher characteristics, relationship to participants, and potential influences | Audit Trail, Supplementary Materials |
| S7 | Context | Describes the study setting/site and relevant contextual factors | Methods |
| S8 | Sampling strategy | Sampling method and saturation criteria | Methods |
| S9 | Ethical issues | Details on ethics approval and consent procedures | Methods |
| S10 | Data collection methods | Describes data collection types and procedures, including iterative aspects and modifications | Methods |
| S11 | Data collection instruments and technologies | Describes instruments used (e.g., interview guides) and any changes made | Methods; Supplementary Materials |
| S12 | Units of study | Number and characteristics of participants and their level of involvement | Methods |
| S13 | Data processing | How data were processed, transcribed, anonymized, and managed | Methods |
| S14 | Data analysis | How themes/inferences were developed, including coding and analysis approach | Methods |
| S15 | Techniques to enhance trustworthiness | Techniques used to enhance credibility (e.g., triangulation, audit trails) | Methods; Supplementary Materials |
| S16 | Synthesis and interpretation | Summary of main themes, inferences, or theoretical developments | Results |
| S17 | Links to empirical data | Use of quotes or other data to support findings | Results |
| S18 | Integration with prior work, implications, etc. | Short Summary of main findings, connection to existing literature, theoretical implications, and study contributions | Discussion |
| S19 | Limitations | Discusses limitations of the study and findings' trustworthiness | Discussion |
| S20 | Conflicts of interest | Disclosure of any conflicts and how they were managed | Competing Interest |
| S21 | Funding | Sources of funding and role of funders | Funding |
