# Supplementary material for: Sociotechnical influences on the adoption and use of AI-enabled clinical decision support systems in ophthalmology: a theory-based interview study
Source: BMC Health Serv Res. 2025 Oct 22;25:1398. doi: 10.1186/s12913-025-13620-w (PMC12542331; doi:10.1186/s12913-025-13620-w)
Supplement: Supplementary file 2 — Additional File 2: English version of the semi-structured interview guide. [file 12913_2025_13620_MOESM2_ESM.docx]

Semi-structured interview guide for participants without own AI-CDSS usage:

*Indented questions were asked optionally, depending on the flow of the interview, relevance to participants’ roles, and available time resources.*

*In interviews with assistants, some questions were phrased slightly differently (e.g., “What would it take for you to welcome the introduction of an ophthalmic AI tool in your everyday work” instead of “*What would it take for you to decide to start using an ophthalmic AI tool in your everyday work?*”).*

[Introduction]

- What tasks do you generally deal with in your day-to-day work?

[Definition AI & AI-CDSS]

- How familiar are you personally with ophthalmic AI tools?
  - For example, have you already encountered an ophthalmic AI tool first-hand?
  - Can you describe this AI tool?
  - To what extent have you personally dealt with / are personally dealing with this AI tool in your day-to-day work?
- What are the reasons why you are currently not routinely using an ophthalmic AI tool for your work?
  - To what extent do you think you would be involved in the decision whether such an AI tool should be introduced at your institution/department?

[Transition]

- What would it take for you to decide to start using an ophthalmic AI tool in your everyday work? Or, in other words, under what conditions would you decide to start using an ophthalmic AI tool?
  - What would prevent you personally from deciding to start using such an AI tool in your daily clinical practice?
- What characteristics would the AI tool itself need to have so that you would consider using it for the first time in your everyday clinical practice?
  - What would the AI tool have to achieve or accomplish so that you would consider using it for the first time in your everyday clinical practice?
  - How would you realize that using the AI tool in your daily work is worthwhile?
  - Would you personally fear any risks from using AI tools in your day-to-day work?
  - Would you say that these concerns would influence your decision whether to start using an AI tool in your daily clinical practice?
- What do you think: How would the introduction of an AI tool in your facility change your work or your day-to-day work routine?
  - Do you think that the introduction of an AI tool would have an impact on your role or how you view yourself as an [profession]? If so, in what way?
  - Would you say that these thoughts would influence your decision to start using an AI tool in your clinical practice?
  - What impact do you think the introduction of an AI tool would have on your workload?
  - What impact do you think the introduction of an AI tool would have on your relationship with your patients?
  - How would these considerations influence your decision to start using an AI tool in your clinical practice?
- What requirements would your facility and the people working there have to fulfill so that you would decide to start using an AI tool?
- Are there any political or sociocultural factors that would influence your decision to start using an AI tool in your day-to-day work?

[Transition]

- Can you now describe a typical workflow from your day-to-day work in more detail (e.g. when dealing with a typical patient)?

[Transition]

- Under what conditions could you effectively integrate such a newly introduced AI tool into your typical workflows? What would be needed?
  - What challenges or problems do you think would arise when trying to effectively integrate an AI tool into your typical workflows?
  - How might these challenges be overcome?
- In your opinion, what requirements would the newly introduced AI tool itself have to fulfill so that you could integrate it effectively into your typical workflows of your clinical work?
- What requirements would your facility, including its technology, physical environment and people, need to fulfill so that you could effectively integrate the AI tool into your typical workflows?
- Do you think that you personally would have to fulfill certain characteristics or other requirements in order to be able to effectively integrate the AI tool into your typical workflows?
- Compared to other medical specialties, do you think that there is anything that needs to be considered when using AI specifically in ophthalmology? Or, in other words, does the field of ophthalmology differ from other medical fields in a way that could be relevant to the development and use of AI tools in everyday work?

[Wrap-up]

- Is there anything you would you like to add?

Semi-structured interview guide for participants with own AI-CDSS usage:

*Indented questions were asked optionally, depending on the flow of the interview, relevance to participants’ roles, and available time resources.*

*In interviews with assistants, some questions were phrased slightly differently (e.g., “What would it take for you to welcome the introduction of an ophthalmic AI tool in your everyday work” instead of “*What would it take for you to decide to start using an ophthalmic AI tool in your everyday work?*”).*

[Introduction]

- What tasks do you generally deal with in your day-to-day work?

[Definition AI & AI-CDSS]

- How familiar are you personally with ophthalmic AI tools?
  - For example, have you already encountered an ophthalmic AI tool first-hand?
  - Can you describe this AI tool?
  - To what extent have you personally dealt with / are personally dealing with this AI tool in your day-to-day work?
- How long have you been using this AI tool?
  - Did you personally experience the introduction of this AI tool at your facility at the time?
  - Who else at your facility is using this AI tool?
  - How did the introduction of this AI tool come about at the time? What led to the decision to introduce this AI tool? Who made this decision?
  - What considerations were made in advance in relation to…
    - The AI tool itself
    - Its expected impact and value
    - The potential users
    - The facility
    - Political or sociocultural factors
- Can you describe what impact the introduction of this AI tool has (had) on your personal day-to-day work?
  - Has the introduction of this AI tool had an impact on your role or how you view yourself as an [profession]? If yes, in what way?
  - What impact did the introduction of the AI tool have on your workload?
  - What impact did the introduction of the AI tool have on the relationship with your patients?
- How would you evaluate your experience with this AI tool so far?
  - Have you noticed any benefits? If yes, in what way?
  - Have you encountered any problems when using the AI tool in everyday clinical practice?
  - How did you deal with these problems?
- What factors facilitated or hindered the integration of the AI tool into your typical workflows?

[Transition]

- What would it take for you to decide to start using another ophthalmic AI tool in your everyday work. Or, in other words, under what conditions would you decide to start using another ophthalmic AI tool?
  - What characteristics would the AI tool itself need to have so that you would consider using it in your everyday clinical practice?
  - What do you think, how would the introduction of another AI tool in your facility change your work or your day-to-day work routine?
  - What requirements would your facility and the people working there have to fulfill so that you would decide to start using another AI tool?
  - Are there any political or sociocultural factors that would influence your decision to start using another AI tool in your day-to-day work?

[Transition]

- Under what conditions could you effectively integrate such a newly introduced AI tool into your typical workflows of your clinical work? What would be needed?
  - What challenges or problems do you think would arise when trying to effectively integrate another AI tool into your typical workflows?
  - In your opinion, what requirements would the newly introduced AI tool itself have to fulfill so that you would be able to integrate it effectively into the typical workflows?
  - What requirements would your facility, including its technology, physical environment and people, have to fulfill so that you could effectively integrate the AI tool into your typical workflows?
  - Do you think that you personally would have to fulfill certain characteristics or other requirements in order to be able to effectively integrate the AI tool into your typical workflows?
  - Compared to other medical specialties, do you think that there is anything that needs to be considered when using AI specifically in ophthalmology? Or, in other words, does the field of ophthalmology differ from other medical fields in a way that could be relevant to the development and use of AI tools in everyday work?

[Wrap-up]

- Is there anything you would you like to add?
