# Supplementary material for: Sociotechnical influences on the adoption and use of AI-enabled clinical decision support systems in ophthalmology: a theory-based interview study
Source: BMC Health Serv Res. 2025 Oct 22;25:1398. doi: 10.1186/s12913-025-13620-w (PMC12542331; doi:10.1186/s12913-025-13620-w)
Supplement: Supplementary file 3 — Additional File 3: Additional table with sample characteristics at the individual participant level. [file 12913_2025_13620_MOESM3_ESM.docx]

**Table 2.** Detailed characteristics of individual participants.

| ID | Gender | Age | Employment status | Role | Years of experience | Country | Facility | AI familiarity | | AI-CDSS usage |
| --- | --- | --- | --- | --- | --- | --- | --- | --- | --- | --- |
|  |  |  |  |  |  |  |  | In general | In opthalmology |  |
| **Ophthalmologists** | |  |  |  |  |  |  |  |  |  |
| O1 | F | 36 | Part time (> 50%) | Attending | 11 | Germany, Switzerland | Group practice | Rather unfamiliar | Not familiar at all | No usage in daily work |
| O2 | M | 63 | Full time | Head of department | 35 | Germany | Specialized hospital for ophthalmology | Rather familiar | Rather familiar | Regular usage for clinical work |
| O3 | M | 25 | Full time | Resident | 6 months | Germany | University-affiliated hospital | Rather familiar | Rather familiar | No usage in daily work |
| O4 | M | 37 | Full time | Head of department | 11 | Austria | General hospital | Rather familiar | Somewhat familiar | Irregular usage for clinical work |
| O5 | F | 36 | Part time (> 50%) | Attending | 11 | Germany | University-affiliated hospital | Rather familiar | Somewhat familiar | Irregular usage for clinical work |
| O6 | M | 44 | Full time | Attending | 17 | Germany | Group practice | Rather familiar | Rather familiar | Unsure if regularly used tool is AI-based |
| O7 | M | 58 | Full time | Head of department | 35 | Germany | General hospital | Somewhat familiar | Somewhat familiar | No usage in daily work |
| O8 | M | 25 | Full time | Resident | 4 | Switzerland | General hospital | Rather familiar | Rather familiar | Research on AI tools, but not used for clinical work |
| O9 | M | 33 | Full time | Resident | 2 | Austria | University-affiliated hospital | Somewhat familiar | Rather unfamiliar | No usage in daily work |
| O10 | F | 37 | Part time (< 50%) | Attending | 10 | Germany | Group practice | Not familiar at all | Not familiar at all | Unsure if regularly used tool is AI-based |
| O11 | F | 70 | Full time | Attending | 40 | Germany | Group practice | Not familiar at all | Somewhat familiar | No usage in daily work |
| O12 | M | 30 | Full time | Resident | 7 | Germany | University-affiliated hospital | Rather familiar | Rather familiar | Regular usage for clinical work |
| O13 | F | 30 | Full time | Resident | 4 | Switzerland | Group practice | Somewhat familiar | Somewhat familiar | Research on AI tools, but not used for clinical work |
| O14 | F | 34 | Full time | Resident | 8 | Germany | University-affiliated hospital | Somewhat familiar | Somewhat familiar | Conducted research on AI in ophthalmology and was unsure whether AI was already integrated into some devices at her facility. |
|  |  |  |  |  |  |  |  |  |  |  |
| **Supporting Ophthalmic Personnel** | | | |  |  |  |  |  |  |  |
| A1 | F | 55 | Full time | Ophthalmic study nurse | 20 | Germany | Group practice | Rather unfamiliar | Rather unfamiliar | No usage in daily work |
| A2 | F | 31 | Full time | Ophthalmic study nurse | 11 | Germany | Group practice | Rather familiar | Rather unfamiliar | No usage in daily work |
| A3 | F | 52 | Part time (> 50%) | Formerly ophthalmic assistant, now practice management consultant | 32 | Germany | Formerly group practice, now consulting firm | Rather unfamiliar | Rather unfamiliar | No usage in daily work |
| A4 | F | 32 | Parental leave | Ophthalmic assistant | 12 | Germany | Group practice | Somewhat familiar | Rather familiar | No usage in daily work |
| A5 | F | 36 | Full time | Optometrist | 3 | Austria | Optical store / optometry practice | Somewhat familiar | Rather unfamiliar | No usage in daily work |
| A6 | F | 64 | Part time (> 50%) | Ophthalmic assistant | 43 | Germany | Group practice | Not familiar at all | Not familiar at all | No usage in daily work |
| A7 | F | 40 | Part time (> 50%) | Ophthalmic assistant | 23 | Germany | Group practice | Rather unfamiliar | Rather unfamiliar | No usage in daily work |
| A8 | M | 57 | Part time (> 50%) | Optometrist | 29 | Switzerland | Optical store / optometry practice | Rather unfamiliar | Rather unfamiliar | Unsure if regularly used tool is AI-based |
