# Supplementary material for: Sociotechnical influences on the adoption and use of AI-enabled clinical decision support systems in ophthalmology: a theory-based interview study
Source: BMC Health Serv Res. 2025 Oct 22;25:1398. doi: 10.1186/s12913-025-13620-w (PMC12542331; doi:10.1186/s12913-025-13620-w)
Supplement: Supplementary file 4 — Additional File 4: Codebook including code definitions, frequencies, and exemplary quotes. [file 12913_2025_13620_MOESM4_ESM.docx]

| **Code** | | **Definition** | **Example Quotes** | **Frequency** |
| --- | --- | --- | --- | --- |
|  | |  |  |  |
| **Familiarity & Experience** | |  |  |  |
| Self-Assessed Familiarity | | This code is used for segments in which the interviewee provides an assessment of how familiar they think they are with AI in ophthalmology. | “No, I actually haven’t really engaged much with AI tools so far—neither privately nor professionally.“ (O10)  “I actually can't say with certainty which of the technical devices we use in the eye clinic don’t already have AI tools integrated in some indirect way.“ (O14) | 23 |
| Points of Contact & Information | | This code is used for segments in which the interviewee describes how they came into contact with ophthalmic AI tools or where they’ve heard or learned about such tools. | “I did attend seminars where this was talked about (…).“ (A5) | 33 |
| Description & Evaluation of Used Tools | | This code is used for segments in which the interviewee describes or evaluates ophthalmic AI-CDSS they have used (e.g., how the tool operates or how it performed). | “In our diabetology unit, there's a device that basically takes fundus photos. These photos are then forwarded—back then they were sent to the U.S.—and analyzed to determine whether the person has diabetes.“ (O5) | 43 |
|  | |  |  |  |
| **Sociotechnical Influences (structured according to the NASSS framework)** | | |  |  |
| *Condition & Use Case* | |  |  |  |
| Suitable Applications | | This code is used for segments in which the interviewee describes ophthalmic diseases, data modalities, clinical settings, or application areas that are deemed suitable for the use of AI. | “I’d use it for complex cases where as many images as possible are taken and then have to be analyzed.” (O1)  “Especially when cases get more complex, I think it’s helpful. When you perhaps don’t know anymore what to do.” (O3) | 34 |
| Unsuitable Applications | | This code is used for segments in which the interviewee describes ophthalmic diseases, data modalities, clinical settings, or application areas that are deemed unsuitable for the use of AI. | “When it comes to things like neuro-ophthalmological cases, I don't think AI is suitable. Because there you need to have talked to the patient and looked closely at even the smallest details. I just can’t imagine AI handling that.“ (O1)  “If it targets the patient interactions, [I’d rather reject the use of AI tools in my practice] because I think this has to be personal.” (A1) | 16 |
| *AI Tool* | |  |  |  |
| Usability | | This code is used for segments in which the interviewee describes the role of usability of ophthalmic AI-CDSS for their adoption decisions and added value of such tools in clinical practice. | “Ideally, it shouldn't take ages to read up on how it works.“ (O3)  “One factor, for example, is how much time the AI needs to generate an analysis. If it takes so long that I could do it faster myself, then it’s not of interest to me. (…) It shouldn’t have too much unnecessary frills. We see it with many tools: There are a thousand parameters being generated, and then you can’t see the forest for the trees.“ (O6) | 93 |
| (Validated) Performance | | This code is used for segments in which the interviewee describes the role of reliability and accuracy of ophthalmic AI-CDSS (e.g., as validated by empirical studies) for their adoption decisions and added value of such tools in clinical practice. | “I think the most important thing is reliability—that it works just as reliably as when the analysis is done by other trained professionals.“ (A2)  “I definitely need to be convinced that the tool delivers valid results. I think it’s absolutely important that this is backed by clinical studies.” (O13) | 52 |
| Relevant Features & Functionalities | | This code is used for segments in which the interviewee describes the role of specific features or functionalities of ophthalmic AI-CDSS for their adoption decisions and added value of such tools in clinical practice (e.g., certain analytic or output options). | “And above all, visualization—that would be brilliant. For example, showing changes at the OCT level.“ (O5) | 27 |
| Data Security | | This code is used for segments in which the interviewee describes the role of data security of ophthalmic AI-CDSS for their adoption decisions and added value of such tools in clinical practice. | “Two things were important to me. First, that no patient data are transmitted. So that the practice’s privacy and the anonymity of patient data are preserved. And second, that if possible, it doesn’t run on some external server, but on our own.“ (O2) | 14 |
| Interoperability | | This code is used for segments in which the interviewee describes the role of interoperability of ophthalmic AI-CDSS for their adoption decisions and added value of such tools in clinical practice. | “And of course, compatibility with the devices that are already there—so that the technology can actually be used with them.“ (O14) | 10 |
| Transparency & Explainability | | This code is used for segments in which the interviewee describes the role of transparency or explainability of ophthalmic AI-CDSS for their adoption decisions and added value of such tools in clinical practice. | “I want the decisions to be traceable, verifiable, that you can perhaps cross-check to a certain extent.“ (O9) | 8 |
| Costs | | This code is used for segments in which the interviewee describes the role of costs of ophthalmic AI-CDSS for their adoption decisions and added value of such tools in clinical practice. | “A reason not to get it would be if it's too expensive to purchase.“ (O10) | 7 |
| Vendor Support Services | | This code is used for statements in which the interviewee describes the role of availability of vendor support services for their adoption decisions and added value of ophthalmic AI-CDSS in clinical practice. | “And I think especially in the beginning, when this technology is newly introduced, there should be a direct contact with the manufacturers or distributors—so that there can be a close exchange about training and any questions that come up.“ (O14) | 5 |
| Regulatory Approval | | This code is used for segments in which the interviewee describes the role of regulatory approval of ophthalmic AI-CDSS for their adoption decisions and added value of such tools in clinical practice. | “Naturally, it would be reasonable for it to be approved, meaning that it was tested and approved as a medical device.“ (O4) | 2 |
| *Value Proposition* | |  |  |  |
| Worthwhile Solution to Unmet Needs | | This code is used for segments in which the interviewee mentions that ophthalmic AI-CDSS would have to address a relevant need, with benefits justifying their costs. The code is also used when interviewees mention that they have not (yet) adopted an AI-CDSS because these do not (yet) seem worthwhile. | “There’s no product on the market that would offer us enough added value that getting it is worth it.“ (O4) | 19 |
| Efficiency Gains | | This code is used for segments in which the interviewee describes the role of increased efficiency of profit for their adoption decisions and added value of ophthalmic AI-CDSS in clinical practice. | “And it would need to reduce waiting times for patients substantially, so that it’s really noticeable.“ (A3) | 28 |
| Facilitation of Work | | This code is used for segments in which the interviewee describes the role of facilitation of work for their adoption decisions and added value of ophthalmic AI-CDSS in clinical practice (e.g., increasing confidence in one's clinical decisions, simplifying tasks, reducing cognitive load, or increasing joy during clinical tasks). | “Well, it would need to make my job easier.“ (A1) | 28 |
| Improvement in Quality of Care | | This code is used for segments in which the interviewee describes the role of improved quality of care for their adoption decisions and added value of ophthalmic AI-CDSS in clinical practice. | “It has to protect me from mistakes, from careless ones. And if possible, it should also provide additional knowledge, because I know I don’t know everything.“ (O2)  “I think there really needs to be a clear benefit for the patients in the end so that you use it. Otherwise, why add this extra step in between?“ (A3) | 18 |
| *Adopters* | |  |  |  |
| General Perceptions on AI in Ophthalmology | General Attitude | This code is used for segments in which the interviewee mentions how they generally think or feel about AI in ophthalmology. | “But I find it really interesting. (...) Exciting topic!“ (O10) | 41 |
|  | AI’s Potential in Ophthalmology | This code is used for segments in which the interviewee describes the potential they think AI has for ophthalmology as a medical specialty. | “I think ophthalmology is very well suited for such AI systems.“ (O11) | 29 |
|  | Status Quo & Future Developments | This code is used for segments in which the interviewee mentions how they perceive the current status and future development of AI in ophthalmology. | “And if things continue to develop the way I predict, and these devices really can support us in that way, it’s only a matter of time before this becomes standard in every practice.” (O6)  “Right now, it’s really just about the analysis. I mean, okay, we don’t know how far this will go. I think in China or America, somewhere they’re already researching some kind of “intravitreal injections robot” that can simply inject the medication into patients. Who knows, no idea. But I think that’s still a long way off.“ (A3) | 27 |
|  | General Risks & Limitations of AI | This code is used for segments in which the interviewee describes general risks and limitations of AI they perceive. | “I see that for many who invest in and research and test AI—as with other areas of research—it’s only about eventually generating profit, financial gain.“ (A8)  “[I fear] that you don’t rely on your own expertise anymore, just on the AI. That you don’t pay attention anymore and check yourself.“ (A5) | 13 |
| Expected Impact on Work | Challenges & Risks | This code is used for segments in which the interviewee describes what challenges, problems, or risks, if any, they expect to occur following the introduction of ophthalmic AI-CDSS in their daily work. | “I wouldn’t see any specific problems at this point—it just needs to be looked at more concretely.“ (A6)  “The only danger is that the training [of the AI model] somehow was not adequate. But I think there are so many safety nets that in the end, no AI will be trained on faulty data. I can’t imagine that.” (O6)  “I think applying [AI] could perhaps be more difficult with older patients, much more difficult than with younger patients who are probably more open to it, I imagine.” (A1) | 55 |
|  | Quality of Care | This code is used for segments in which the interviewee describes what changes in the quality of care, if any, they expect to occur following the introduction of ophthalmic AI-CDSS in their daily work. | “Improving the usefulness of all these examination results and so on. That’s something I could imagine, yes.“ (A6) | 15 |
|  | Working Conditions | This code is used for segments in which the interviewee describes what changes in their working conditions, if any, they expect to occur following the introduction of ophthalmic AI-CDSS in their daily work. | “I would expect it to provide relief. Especially when it comes to decision-making, probably.“ (A1)  “I actually think that if AI allows to see and treat patients more quickly, the consequence will more likely be that more patients are scheduled, not that the workload will somehow decrease.” (O3) | 47 |
|  | Tasks & Professional Identity | This code is used for segments in which the interviewee describes what changes in their tasks, workflows, role, or professional identity, if any, they expect to occur following the introduction of ophthalmic AI-CDSS in their daily work. | “I think using AI will simply change our work a bit, but our role will likely remain the same.” (O13) | 56 |
|  | Patient Relationships | This code is used for segments in which the interviewee describes what changes in their patient relationships, if any, they expect to occur following the introduction of ophthalmic AI-CDSS in their daily work. | “Sure, it’s possible that you might not take as much time for the patients, but I think that when you are talking to them—that also depends on the doctor’s personality, of course—but I think that even in less time, you can still address all questions.“ (O10) | 19 |
| Required Adopter Characteristics | Adoption Readiness | This code is used for statements in which the interviewee describes the role of their own adoption readiness for their adoption decisions and added value of ophthalmic AI-CDSS in clinical practice (e.g., their openness, acceptance, willingness to learn, interest, flexibility). | “I think—as with anything—you have to be open to it. I believe that’s the most important thing.“ (A1) | 21 |
|  | Adequate Usage & Knowledge | This code is used for statements in which the interviewee describes the role of adequate usage (e.g., calibrated trust, usage as intended) and knowledge (e.g., technical or ophthalmic knowledge) for their adoption decisions and added value of ophthalmic AI-CDSS in clinical practice. | “You need to be able to operate the device, know what to watch out for and how it works.“ (A4) | 34 |
| *Organization* | |  |  |  |
| Infrastructure & Resources | Technology & Digitalization | This code is used for statements in which the interviewee describes the role of their facility’s technological and digital infrastructure for their adoption decisions and added value of ophthalmic AI-CDSS in clinical practice. | “The issue is processing power and system stability—most computers aren’t designed for that. That means, you need a specialized machine, and the software must run reliably. So you need a certain infrastructure.“ (O12)  “The second issue was that you always had to manually connect to a secure channel, which wasn’t permanent and couldn’t be established automatically by the machine.” (O2) | 26 |
|  | Workforce | This code is used for statements in which the interviewee describes the role of their facility’s workforce characteristics for their adoption decisions and added value of ophthalmic AI-CDSS in clinical practice (e.g., workforce openness and qualifications). | “It could also mean that additional staff is needed—that we’d have to hire someone new, whether in an administrative, medical, or nursing role.“ (O4)  “I find it even harder to imagine [introducing an AI tool into my practice] with the nurses I currently have; they’re a bit older. (…) That could be a problem, if they decide to switch to another practice or retire. Then we’d have to hire younger staff who can familiarize themselves with [AI tools] more easily.” (O1) | 24 |
|  | Support | This code is used for statements in which the interviewee describes the role of their facility’s support infrastructure for their adoption decisions and added value of ophthalmic AI-CDSS in clinical practice. | “There would definitely need to be a technician available. I’ve always found it a bit difficult when there’s a problem with the device and you end up stuck on the hotline and so on. So it would really make sense to have someone on site who can step in if something’s not working, to get it back up and running quickly.“ (O10) | 13 |
| Administrative & Financial Barriers | | This code is used for segments in which the interviewee describes the role of administrative or financial barriers for their adoption decisions and added value of ophthalmic AI-CDSS in clinical practice (e.g., strict data protection rules, slow processes, reluctant decision-makers). | “In the clinic, I would’ve had to deal with a ton of administration. Getting anything moving there takes time.“ (O2)  “I'm employed. I have the devices my employer provides. And I strongly assume that if I came back from a conference and said, ‘Now I need this’, the answer would be ‘No’. For cost reasons alone.“ (O1) | 15 |
| Training & Communication | | This code is used for segments in which the interviewee describes the role of training or organizational communication for their adoption decisions and added value of ophthalmic AI-CDSS in clinical practice. | “During the onboarding, I always find it important, that it’s introduced well and its use is clearly explained, so that you can actually use it.“ (A2)  “That there’s proper onboarding for all medical staff—not just the doctors, but also the assistants.” (O8) | 42 |
| Workflow Adaptations & Implementation Strategy | | This code is used for segments in which the interviewee describes necessary changes in workflows or the role of a strategic implementation for their adoption decisions and added value of ophthalmic AI-CDSS in clinical practice. | “Any product that is introduced needs a plan, a process. In most cases, workflows will need to be adjusted in some way.“ (O4)  “AI applications really need to be included in the hospital’s overall digitalization strategy, not just at the ophthalmology department.” (O8) | 33 |
| Physical Environment | | This code is used for segments in which the interviewee describes the role of their facility’s physical environment for their adoption decisions and added value of ophthalmic AI-CDSS in clinical practice. | “It would need to be easily accessible somehow. (…) It can’t be that I have to walk through the whole practice just to get to the one room where the AI module is installed. It needs to be easily accessible for all staff who need to use it—probably at multiple locations.“ (A1) | 7 |
| *Wider System* | |  |  |  |
| Healthcare Politics & Regulation | | This code is used for segments in which the interviewee describes the role of political decisions and the regulatory landscape for their adoption decisions and added value of ophthalmic AI-CDSS in clinical practice. | “What makes things easier is that we’re a university hospital, and we do research, which gives us certain freedoms, as long as it's applied within a research context. But on the other hand, what makes it harder is that you really need to know the legal framework in order to apply something like this. The regulations are very strict, and I’d say integration is almost impossible outside of a university setting—unless it’s a commercial product, which is very expensive.“ (O12)  “I think that ties into the issue of regulatory approval, that you definitely need to have assurance that these AI tools are proven to deliver reliable diagnostics or make sound decisions.“ [O13] | 17 |
| Healthcare System Characteristics | | This code is used for segments in which the interviewee describes the role of healthcare system characteristics for their adoption decisions and added value of ophthalmic AI-CDSS in clinical practice (e.g., extent of digitalization, possibility of telemedicine). | “We’re at a point in our digitalization where we can put prescriptions on the health insurance card. What AI? That‘s not true for everyone, of course, but here in Germany, it took a huge effort just to get that implemented.“ (O1) | 8 |
| Public & Patients’ Perception of AI | | This code is used for segments in which the interviewee describes the role of public or patient perceptions for their adoption decisions and added value of ophthalmic AI-CDSS in clinical practice. | “There simply needs to be acceptance of this technology—within society, within internal hospital policies, and also in external health politics. (…) That society and patients are also accepting of it and probably that the overall perception of AI is rather positive.“ (O14) | 10 |
| Reception within Medical Community | | This code is used for segments in which the interviewee describes the role of the medical community’s reception of (ophthalmic) AI tools for their adoption decisions and added value of ophthalmic AI-CDSS in clinical practice. | “Of course, it would be desirable to have a reference clinic that already has experience with this tool—somewhere we could see how it’s been implemented because that facilitates the introduction and give us confidence that it actually delivers on what it promises.“ (O4) | 22 |
| *Embedding & Adaptations Over Time* | |  |  |  |
| Decision Process | Initial Tests & Explorations | This code is used for segments in which the interviewee mentions that exploring and testing available AI tools would precede—or has preceded—their adoption decision. | “I’ve looked at various tools. (…) I’m currently in the process of integrating AI tools, now that I’ve completed the testing phase and know that it works.“ (O2) | 9 |
|  | Decision-Makers & Process | This code is used for segments in which the interviewee describes the institutional adoption decision process and involved decision-makers. | “So probably the department head would be the primary decision-maker. I imagine it would most likely be introduced in the outpatient department first, and there, it would probably be up to the senior physicians responsible for that area to decide whether it makes sense for them.“ (O3) | 17 |
| Usage Trajectories | Familiarization & Adaptations | This code is used for segments in which the interviewee describes the process of establishing and familiarizing oneself with a newly introduced ophthalmic AI-CDSS, including re-evaluations and adaptations. | “And then maybe also meet regularly over time and evaluate whether everything is working the way it should.“ (O3) | 10 |
|  | Evaluation of Added Value & Abandonment | This code is used for segments in which the interviewee describes how they might abandon ophthalmic AI-CDSS over time if it does not provide sufficient value or is too burdensome to use. | “If it ultimately doesn’t pay off adequately (…) and ends up placing a greater financial burden on the institution using it (…), those would be criteria that would need to be examined before considering continued use.“ (O14)  **“A**s soon as it becomes inconvenient or essentially cumbersome, we’ll abandon it.**“ (O6)** | 8 |
|  | Building Trust | This code is used for segments in which the interviewee describes a gradual trust building process, promoted by witnessing the accuracy or benefits of the newly introduced tool. | “Then you would try it out yourself and see whether it aligns with your own assessments, or let it weigh in on simpler cases and double-check yourself, so to say. And if that works out, I think that would reduce my concerns.“ (O9) | 10 |
|  | Overreliance | This code is used for segments in which the interviewee describes a risk of overreliance on ophthalmic AI-CDSS with their use over time. | **“It’s also imaginable that the device could generate the treatment plan itself, and all you'd need to do is give your approval. That raises the question of whether you’d still be as thorough, or if you'd fall into a pattern of just saying 'yes, yes, yes' and end up overlooking something. So that could actually be a trap.“ (O10)** | 5 |
