# Supplementary material for: Sociotechnical influences on the adoption and use of AI-enabled clinical decision support systems in ophthalmology: a theory-based interview study
Source: BMC Health Serv Res. 2025 Oct 22;25:1398. doi: 10.1186/s12913-025-13620-w (PMC12542331; doi:10.1186/s12913-025-13620-w)
Supplement: Supplementary file 5 — Additional File 5: Additional table with a description and evaluation of the AI-CDSS that participants had used for their clinical work. [file 12913_2025_13620_MOESM5_ESM.docx]

**Table 3.** Overview of ophthalmic AI-CDSS used by participants in clinical practice.

| ID | Imaging modality | Purpose | Origin | Evaluation |
| --- | --- | --- | --- | --- |
| O12 | Anterior Segment OCT | Quantify the graft detachment area after Descemet Membrane Endothelial Keratoplasty | Developed as part of an in-house research project | The tool enabled higher precision and usefulness of OCT images.  When functioning properly, it supported treatment quality, but occasional malfunctions, high computational demands, and programming errors caused some delays and extra burden. |
| O4 | OCT | Analyze and quantify retinal features (e.g., fluid, atrophies) and provide treatment recommendation for wet AMD | Tool was available as part of a study | The AI tool was still in its early stages, requiring frequent manual corrections and deviations from protocols to ensure patient safety. However, it demonstrated significant potential to reduce workload once fully developed. |
| O5 | Fundus photography | Diagnose diabetes | Tool was available as part of a study | The participant used the tool only once. No evaluation expressed. |
|  | OCT | Not mentioned. | Commercial product | So far, the tool created more workload than it reduced, but the participant expects it to facilitate work in the long run. |
| O2 | Fundus photography | Identify regions of interest | Commercial product | The tool provided a valuable second opinion, which helped to avoid overlooking important details and boosted confidence, particularly in solo practice settings with high time constraints. Uploading the images, however, required several additional manual steps, which reduced the tool’s usability. |

*Note*. This overview includes only accounts of participants who were sure that the tool in use was AI-based.
